# The Lethal Connection: Investigating the Relationship of Drought Conditions on Firearm and Nonfirearm Suicides Among U.S. Adults

**DOI:** 10.1029/2025GH001571

**Published:** 2026-05-19

**Authors:** Azar M. Abadi, Yeongjin Gwon, Melissa J. Smith, Jesse D. Berman, Austin Rau, Ronald D. Leeper, Jared Rennie, Siddhi Munde, Babak J. Fard, Jesse E. Bell

**Affiliations:** ^1^ Department of Environmental Health Sciences School of Public Health University of Alabama at Birmingham Birmingham AL USA; ^2^ Department of Biostatistics College of Public Health University of Nebraska Medical Center Omaha NE USA; ^3^ Department of Biostatistics School of Public Health University of Alabama at Birmingham Birmingham AL USA; ^4^ Division of Environmental Health Sciences School of Public Health University of Minnesota Minneapolis MN USA; ^5^ North Carolina Institute for Climate Studies North Carolina State University Raleigh NC USA; ^6^ NOAA's National Centers for Environmental Information Asheville NC USA; ^7^ Department of Environmental Agriculture Occupational and Health College of Public Health University of Nebraska Medical Center Omaha NE USA; ^8^ University of Nebraska Daugherty Water for Food Global Institute Lincoln NE USA; ^9^ School of Natural Resources University of Nebraska‐Lincoln Lincoln NE USA

**Keywords:** drought, suicide, firearms, mental health, rural health, environmental epidemiology

## Abstract

Drought is one of the most widespread and disruptive natural hazards globally, with environmental and societal effects that may increase psychological distress. Yet, its association with suicide in the U.S. remains understudied. We examined the relationship between drought and suicide mortality across the contiguous U.S. Drought severity was measured using the Evaporative Demand Drought Index, and suicide data from the National Center for Health Statistics. We used Generalized Additive Models (GAMs) to estimate incidence rate ratios (IRRs) and absolute risk differences (ARDs), with 95% confidence intervals. Analyses were stratified by age, sex, and urbanicity. From 2000 to 2018, the U.S. recorded 350,434 firearm‐related and 323,225 non‐firearm suicide deaths. Drought affected 37.4% of county‐months, and both suicide types were positively associated with drought—especially under severe conditions. For firearm suicides, worsening drought was linked to an IRR of 1.109 (95% CI: 1.091–1.128) and ARD of 0.704 (95% CI: 0.595–0.811); improving drought had an IRR of 1.094 (95% CI: 1.076–1.112) and ARD of 0.608 (95% CI: 0.501–0.715). For non‐firearm suicides, worsening drought was associated with an IRR of 1.057 (95% CI: 1.037–1.077) and ARD of 0.347 (95% CI: 0.232–0.461), while improving drought had an IRR of 1.073 (95% CI: 1.054–1.093) and ARD of 0.456 (95% CI: 0.344–0.568). Severe drought was associated with higher suicide mortality, including firearm‐related deaths, across several subgroups such as older adults, women, and individuals living in non‐metro areas; these subgroup‐specific findings were not statistically compared.

## Plain Language Summary

This study explores how drought, a prolonged period of unusually dry weather, affects suicide rates in the United States. Using data from all counties between 2000 and 2018, we analyzed over 670,000 deaths by suicide to determine if dry conditions were linked to higher risk. We found that both firearm and nonfirearm suicides increased during all stages of drought, with the highest risk seen during the most severe drought periods. Certain groups—including older adults, women, and people living in rural areas—were more affected. The risk was especially high for suicides involving firearms. These findings suggest that dry weather may create or worsen stress, especially for people who are already vulnerable. By better understanding when and where these risks increase, we can improve public health strategies. Adding mental health support to drought preparation and response plans could help save lives, particularly in rural communities and during times of severe environmental stress.

## Key Points

- Firearm and nonfirearm suicides in the U.S. increase during all drought phases, especially during severe drought conditions
- Associations between drought and suicide mortality were observed across subgroups, including older adults, women, and rural residents
- Drought‐related suicide risk underscores the need for mental health integration into early warning and mitigation efforts

## 1 Introduction

Suicide is a major cause of death in the U.S. and has been steadily on the rise since 2008 (Hedegaard, 2021). The increase in suicide risk is not evenly distributed among the total population and some population strata are more at‐risk including children, young adults, aging populations, males, and rural communities (Martínez‐Alés, Pamplin, et al., 2021). Suicide risk is a complex, multifaceted problem that is influenced by a broad spectrum of elements, including but not limited to psychological, socioeconomic, and environmental factors (Kennedy et al., 2021). Climate extremes can exacerbate stressors on livelihoods and induce community‐wide socioeconomic challenges, leading to increased vulnerability and mental health strains (Vins et al., 2015). Extreme climate events can directly and indirectly affect mental health through acute climate‐induced disasters such as floods, wildfires, hurricanes, which disrupt communities and livelihoods, amplifying stress and potentially leading to increased suicide risk (Hwong et al., 2022; Thompson et al., 2023). Furthermore, subacute climate extremes such as long droughts or elevated temperature periods can foster a sense of loss and nostalgia for a changing environment, known as solastalgia, further impacting mental well‐being and potentially increasing the risk of suicide (Hwong et al., 2022; Lynch et al., 2020; Thompson et al., 2023).

Drought is an extreme weather event defined by an extended period of below normal rainfall, which can result in adverse environmental and sociological disruptions, including elevated air pollution, degraded water quality, loss of livelihood, and forced migrations (Abadi et al., 2022; Lynch et al., 2020; Mishra & Singh, 2010; Padrón‐Monedero et al., 2024) Over the past 50 years, drought has killed more people globally than any other climate and weather‐related disaster (Weather‐Related Disasters Increase over Past 50 Years, Causing More Damage but Fewer Deaths, 2021). In the United States, drought is estimated to be the third costliest climate event, with $367.6 billion in losses between 1980 and 2024—ranking just behind tropical cyclones and severe storms (NOAA National Centers for Environmental Information (NCEI) U.S. Billion‐Dollar Weather and Climate Disasters, 2025). According to the same report, it is also the second leading cause of natural disaster‐related deaths in the U.S., with 4,658 lives lost during the same period. However, these figures likely underestimate the true toll, as it is difficult to directly attribute mortality to drought conditions. Many drought‐related deaths occur indirectly, often through secondary hazards such as dust exposure and extreme heat events that frequently co‐occur with drought conditions and may exacerbate drought‐related health risks.

Numerous studies, especially from Australia, have linked droughts to mental health distress like depression, anxiety, and suicide, especially among farmers and their communities (Austin et al., 2018; Berman et al., 2021; Comtesse et al., 2021; OBrien et al., 2014; Stanke et al., 2013; Vins et al., 2015). Longer‐lasting droughts have been observed to adversely affect life satisfaction (Carroll et al., 2009; Luong et al., 2021; OBrien et al., 2014). Studies indicate that age significantly influences suicide rates with children, middle‐aged, and elderly populations exhibiting a heightened risk (Boyd et al., 2007; Carnie et al., 2011; J. G. Dean & Stain, 2010; J. Dean & Stain, 2007; Sartore et al., 2005; Sewell et al., 2024). Factors such as hopelessness, limited adaptive capacity, and easy access to firearms contribute to vulnerability among younger individuals (Boyd et al., 2007; Carnie et al., 2011; Schnitzer et al., 2019). Middle‐aged and older populations face risks due to poor physical health and limited ability to adapt to environmental change or hardship (Daghagh Yazd et al., 2019; Guiney, 2012; Hanigan et al., 2012; Kennedy et al., 2021; Luong et al., 2021; Polain et al., 2011; Santos et al., 2021). Drought affects mental health differently between sexes. Men are more likely to commit suicide due to factors such as job loss, traditional masculine paradigms, reluctance to seek help, and using more violent methods in suicide attempts (Payne et al., 2008). Other studies have shown females show greater mental distress during drought conditions due to their overwhelming burden of being both producer and provider (Coêlho et al., 2004; Hanigan et al., 2018; Kennedy et al., 2020, 2021; Perceval et al., 2019). Urbanization may modify the association between drought and suicide risk due to stark differences between metropolitan and nonmetropolitan settings in terms of economic structure, mental health service availability, infrastructure, and social support systems. Rural and farming communities, in particular, may be more vulnerable to these impacts, as they often face compounding social, cultural, geographical, financial, and occupational challenges that can heighten the risk of adverse mental health outcomes during environmental stressors like drought (Berman et al., 2021; Daghagh Kalesan et al., 2020; Kennedy et al., 2021; Yazd et al., 2019).

The literature on drought and mental health in the U.S. is nascent. While rich in detail, the few existing case studies are limited and non‐generalizable due to their small sample sizes, focused populations (e.g., farmers, older male adults, and children), limited geographic scope, or short study periods (Barreau et al., 2017; Berman et al., 2021; Choudhary & Vaidyanathan, 2014; Sewell et al., 2024). Moreover, these studies rarely examine cause‐specific suicide outcomes, despite the fact that firearm‐related suicide accounts for the majority of suicide deaths in the U.S. and poses unique risk factors related to access, lethality, and sociocultural context. Although international studies—particularly from Australia—have documented associations between drought and mental health issues, including suicide, there is a critical gap in understanding how drought influences firearm and non‐firearm suicide risk in the United States. Our study addresses this gap by estimating the association between drought conditions and suicide incidence using a national, multi‐year, cause‐specific mortality data set. The size and geographic coverage of our data support an assessment of how drought‐related suicide risk varies by method (firearm vs. non‐firearm), as well as by age, sex, and urbanicity—factors that remain underexplored in the current literature.

## 2 Materials and Methods

### 2.1 Outcome

We obtained restricted mortality data from the Centers for Disease Control and Prevention National Center for Health Statistics (NCHS) from 2000 to 2018. Firearm and nonfirearm suicides were identified using the (International Classification of Diseases, 10th Revision) [ICD‐10] codes of X72‐74 for firearm and X60‐69 (Poisoning), X70 (Suffocation), X71 (Drowning), X75, X81 (Other specified, classifiable), X76‐77 (Fire or hot object or substance), X78 (Cut or pierce), X79 (Struck by or against), X80 (Fall), X82 (All transport), X83, Y87.0 (Other specified, not elsewhere classified), and X84 (Unspecified) for nonfirearm mechanism, respectively. We aggregated total suicide deaths by county per month and stratified counts into two adult age groups: 20–64 and 65+ and two sex groups (male, female). County‐level Federal Information Processing Standards (FIPS) codes were used to ensure spatial consistency over time, and known county name changes or jurisdictional reorganizations during the study period were harmonized (e.g., reassignment of Shannon County, SD to Oglala Lakota County, SD, and merging of Bedford city, VA with Bedford County, VA) to prevent artificial discontinuities in mortality time series. Although broad race categories (White, Black, Other) were reported, we did not perform a race analysis as the small population sizes in many race‐county‐year strata precluded us from obtaining stable estimates. Furthermore, attempts to combine non‐White race categories into a single group did not resolve the issue of model convergence. Therefore, to maintain the integrity of our analysis, we ultimately decided to exclude race as a variable. To estimate rates, we assigned annual population estimates from the National Cancer Institute Surveillance, Epidemiology, and End Results (SEER) for the overall population and for the age and sex subgroups. This study was approved by the institutional review board at the University of Nebraska Medical Center and granted exemption as not human subject research (45CFR46.102).

### 2.2 Environmental Exposure

Drought indices are quantitative measures of the relative atmospheric or terrestrial dryness. The Evaporative Demand Drought Index (EDDI) quantifies drought signals through the response of atmospheric evaporative demand (E0) to surface drying anomalies at different timescales to capture drying dynamics (Hobbins et al., 2016; McEvoy et al., 2016). Positive EDDI values indicate conditions drier than normal and negative values indicate wet anomalies. To create interpretable categories of drought, we binned EDDI values into percentile categories of neither wet or dry, D0‐D4 (Abnormally Dry, Moderate Drought, Severe Drought, Extreme Drought and Exceptional Dry), and W0‐W4 (Abnormally Wet, Moderate Wet, Severe Wet, Extreme Wet, and Exceptional Wet). These categories are based on the U.S. Drought Monitor, an influential drought monitoring system in North America. We limited our analysis to medium to longer term droughts (6, 12‐month timescales), as these are more likely to be related to mental health outcomes compared to shorter‐term droughts that may not represent a true persistence of dry conditions (J. G. Dean & Stain, 2010; OBrien et al., 2014). The primary focus of this report is on the 12‐month results. The 6‐month results are detailed as a secondary analysis in the supplementary materials (Table S1 in Supporting Information S1).

Because the 12‐month EDDI represents an aggregation of atmospheric evaporative demand anomalies over a 12‐month moving window, it is a highly smoothed metric; consequently, abrupt transitions between distant drought categories (e.g., from D1 to D4) within a single reporting period (e.g., 1 month) are statistically improbable, typically occurring only under extreme or exceptional atmospheric forcing events. We defined drought events as periods of D1–D4 drought lasting at least two consecutive months to ensure persistence and avoid short‐lived anomalies that are unlikely to have measurable effects on mental health. To assess potential differences in psychological stress across the drought lifecycle, we formulated a Cumulative Drought Intensity (CDI) index using the approach detailed in NOAA's Annual 2020 National Climate Report, multiplying severity level (e.g., 4 for D4) by duration (e.g., 1 month) (NOAA National Centers for Environmental Information, Monthly National Climate Report for Annual, 2020, 2021). CDI was computed as a running cumulative sum of monthly drought severity scores within each drought event and reset to zero when drought conditions ended. This approach captures the combined effect of both severity and duration, which are critical dimensions of exposure likely to shape mental health impacts.

**Figure 1:**
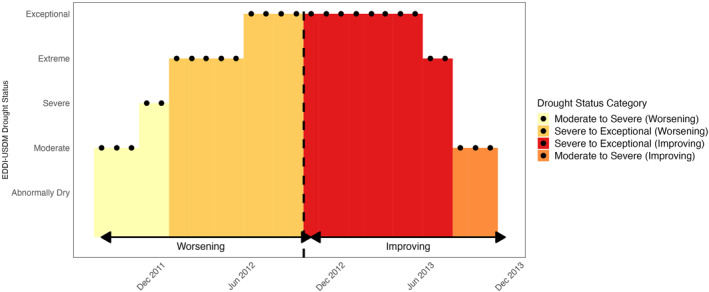
An example of a drought event profile using data from Boone County, NE (2012–2013). Using the 50% of the CDI index, we defined the worsening and improving stages of the drought. The period that falls between moderate‐to‐severe and severe‐to‐exceptional drought in the worsening part of drought are moderate‐to‐severe worsening, and severe‐to‐exceptional drought, respectively. The third stage is the period that falls between severe‐to‐exceptional in the improving part of drought and the last stage is the period that falls between moderate‐to‐severe at the end of the drought event.

We then split each drought event at the point where cumulative CDI reached 50% of the total event CDI, designating the first half as the “worsening” phase and the second half as the “improving” phase. This split is based on cumulative intensity rather than elapsed time; consequently, months with higher drought severity contribute disproportionately to CDI and may dominate one phase of the event. This approach reflects theoretical models of anticipatory versus recovery‐related stress and allows us to examine whether the mental health burden differs depending on whether a drought is intensifying or receding. Monthly drought exposure was defined by combining drought phase (worsening vs. improving) with contemporaneous drought severity. Specifically, each drought month was categorized as (a) worsening moderate‐to‐severe (D1–D2), (b) worsening severe‐to‐exceptional (D3–D4), (c) improving severe‐to‐exceptional (D3–D4), or (d) improving moderate‐to‐severe (D1–D2) (Figure 1). All other months were classified as “None” or “Wet.” We also calculated mean monthly temperature (°C) using the NOAA NClimGrid data set (5 km resolution), applying zonal averaging within county boundaries (Vose et al., 2014).

### 2.3 Urbanization Classes

We characterized counties based on their urbanicity using the NCHS urban‐rural classification schemes to align with temporal changes in county classification. Specifically, we applied the 2006 NCHS scheme to the years 2000–2009 and the 2013 scheme to the years 2010–2018. For the main analysis, we included all counties, regardless of changes in urbanization status. As a sensitivity analysis, we re‐ran models excluding counties whose classification changed between the two periods (113 counties). The estimates from the sensitivity analysis were not significantly different from those of the main model suggesting that the results are robust to the exclusion of counties with changes in urbanization classification. Metropolitan counties encompassed NCHS categories such as large central metro, large fringe metro, medium metro, and small metro. In contrast, nonmetropolitan (non‐metro) counties were those labeled as micropolitan and noncore (Ingram & Franco, 2014).

### 2.4 Statistical Analysis

We generated a national‐level monthly data set at the county‐level by merging suicide mortality counts, EDDI derived drought conditions, monthly mean temperature, and urbanization classes from January 2000 to December 2018 (228 months). We further incorporated a time variable ranging from 1 to 228 for each county's time series, representing the relative position of observations in time. We employed a two‐stage time‐series modeling approach commonly used in environmental epidemiology (Anderson & Bell, 2009; Berman et al., 2019; Dominici et al., 2006). First, we estimated county‐specific associations between drought and suicide incidence using Generalized Additive Models (GAMs) with a negative binomial distribution to account for overdispersion in count data. The use of a GAM supports a flexible non‐linear relationship, including seasonal and long‐term temporal trends (T.J. Hastie & Tibshirani, 1990). The exposure was our categorical variable for drought exposure with “None” as reference level. For each county, we modeled firearm and non‐firearm suicides separately. Models adjusted for season and included smooth functions for monthly mean temperature and time to account for seasonal and long‐term temporal trends, respectively. A log‐transformed population offset was used to model rates. We evaluated spline sensitivity by systematically varying the number of knots for both temperature (*k* = 2, 3, 4, 5) and time (*k* = 3, 4, 5, 6), resulting in 16 total combinations tested per county to assess model stability. Each configuration was applied to model county‐specific data, and the model's adequacy was determined by calculating the Akaike Information Criterion (AIC) (Akaike, 1992). The combination yielding the lowest AIC indicated the most suitable model fit for that particular county. After modeling all counties, we aggregated the results to identify the most frequently optimal knot combination across the nation. Remarkably, the 4‐4 combination (4 knots for temperature and 4 for time) emerged as the most prevalent across US, suggesting it provided the best balance of model complexity and goodness of fit for the majority of counties. This methodology not only underscores the importance of tailored model configurations based on local data characteristics but also enhances the generalizability of our findings by systematically comparing across a diverse set of geographical locations. In the second stage, we applied random‐effect meta‐analysis to combine county‐specific estimates calculated in the first stage to estimate the overall association between drought and both firearm and nonfirearm suicides (Rau et al., 2023). Rather than applying a population threshold, we refined our inclusion criteria by excluding estimates with a standard error greater than 2 to focus on counties with stable IRR estimates in our analysis (Gwon et al., 2023). This exclusion led to the removal of certain counties, which are mapped and detailed in the supplementary material. To assess the robustness of this approach, we conducted a sensitivity analysis comparing results from the standard error threshold (SE ≤ 2) to alternative population‐based thresholds (counties with population ≥10,000, ≥25,000, and ≥30,000). The estimated IRRs remained consistent across these scenarios for both drought constructs (M2SDImp and M2SDWrs), supporting our decision to adopt the SE‐based criterion. Notably, the SE ≤ 2 criterion excluded fewer counties than population‐based thresholds, preserving a larger portion of the national data in the meta‐analysis. Full results are presented in Table S2 in Supporting Information S1. To detail exclusion in the meta‐analysis, we labeled our plots with “Represents over 85% of counties.” or “Represents under 85% of counties.” This approach allows for a clear understanding of the county coverage and potential limitations of our findings.

We conducted separate analysis for firearm and nonfirearm suicides and estimated effects for the overall population and stratified subpopulations by age group (20–64, 65+), sex (male/female), and urbanicity (metro/non‐metro). Models were run for the 6, 12‐month drought timescales representing medium and long‐term droughts, respectively. Results are reported as Incidence Rate Ratios (IRR) with 95% CIs for the association between drought categories and suicide rates, where no drought periods (“None”) is the reference exposure. We additionally calculated the Absolute Risk Differences (ARD) to assess the absolute change in risk attributable to the exposure. This calculation was performed following established methodologies previously detailed in the literature (Di et al., 2017; Rau et al., 2023). Briefly, ARD was derived by combining the relative effect estimate with the baseline suicide rate, and represents the additional number of suicide cases attributable to drought per million persons. Specifically, ARD was calculated as ARD = *α* × (IRR − 1)/IRR, where α denotes the baseline suicide rate in the corresponding population stratum. Confidence intervals for ARD were obtained by propagating uncertainty from the IRR estimate, following established methods. The ARD metric provides a direct measure of the number of additional cases of the outcome associated with the exposure or intervention (per million persons). Statistical analyses were conducted using *R* software (version 4.4.1), using “gam” function from “mgcv” and “rma” function from “metafor” packages.

## 3 Results

### 3.1 Descriptive Statistics

Our primary analysis focused on the 12‐month drought timescale, which represents longer droughts, with the 6‐month timescale reported in the supplementary material (Table S1 in Supporting Information S1). From 2000 to 2018, we identified 350,434 firearm and 323,225 nonfirearm suicides in the contiguous United States (Table 1). Our data showed 75.18% of firearm suicides and 89.29% of nonfirearm suicides were committed by adults aged 20–64. In contrast, adults aged 65 and above accounted for 24.82% of firearm suicides and 10.71% of nonfirearm suicides. This indicates that while a smaller percentage of older adults engage in suicidal acts, they have a relatively higher propensity to choose firearms compared to nonfirearm methods. The data also reveals that males constitute a higher percentage of both firearm (86.77%) and nonfirearm (69.9%) suicides compared to females. Females accounted for 13.23% of firearm suicides and 30.1% of nonfirearm suicides. The distribution of suicide methods varies between metropolitan and nonmetropolitan counties. Metropolitan counties have a higher proportion of both firearm (76.65%) and nonfirearm (85.09%) suicides, compared to nonmetropolitan counties, which account for 23.35% of firearm and 14.91% of nonfirearm suicides. In general, almost half of both firearm (54.48%) and nonfirearm suicides (53.77%) occurred during climate conditions without drought. By contrast, 39% firearm and 39.5% nonfirearm suicide mortalities occurred during drought conditions. It is important to note that within these figures, just over 10% in each category occurred during conditions classified as severe‐to‐exceptional drought, with this percentage representing an aggregate of instances from both worsening and improving severe drought categories as detailed in the table. In our study period, we observed more than half of the county‐months (56.15%) experienced no drought conditions. Almost a third of the county‐months were in moderate‐to‐severe drought (27.63%) and 9.75% in severe‐to‐exceptional drought conditions.

**Table 1:** Baseline Characteristics of the Suicide Mortality in Adult Population and Drought Exposure for Contiguous US (2000–2018)

Category	Suicide deaths			
	Variable	Firearm (Total = 350,434)	Nonfirearm (Total = 323,225)	Frequency (County‐Months)
Person‐based	**Age** (years)			
	20–64	263,442 (75.18%)	288,611 (89.29%)	–
	65+	86,992 (24.82%)	34,614 (10.71%)	–
	**Sex**			
	Male	304,064 (86.77%)	225,947 (69.90%)	–
	Female	46,370 (13.23%)	97,278 (30.10%)	–
Place‐based	**Urbanicity**			
	Metro	268,617 (76.65%)	275,018 (85.09%)	–
	Nonmetro	81,817 (23.35%)	48,207 (14.91%)	–
Exposure	**Drought**			
	None	189,690 (54.48%)	172,041 (53.77%)	397,666 (56.15%)
	Moderate to Severe Worsening	44,652 (12.82%)	41,651 (13.02%)	89,825 (12.68%)
	Severe to Exceptional Worsening	17,683 (5.08%)	16,042 (5.01%)	33,258 (4.70%)
	Severe to Exceptional Improving	18,694 (5.37%)	17,443 (5.45%)	35,747 (5.05%)
	Moderate to Severe Improving	54,774 (15.73%)	51,249 (16.02%)	105,913 (14.95%)
	**Wet Periods**	22,681 (6.51%)	21,552 (6.74%)	45,867 (6.47%)

### 3.2 Association of Droughts With Firearm and Nonfirearm Suicides

**Figure 2:**
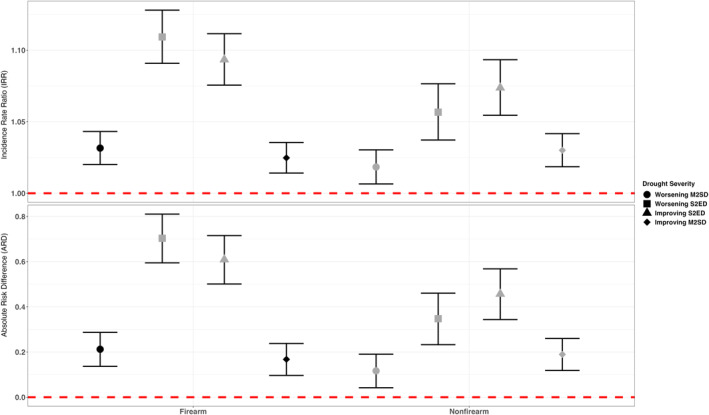
Incidence Rate Ratio (IRR) and the Absolute Risk Difference (per million persons) of firearm and nonfirearm suicides in different drought stages compared to no drought conditions for the total population (M2SD—moderate‐to‐severe Drought, S2ED—Severe‐to‐Exceptional Drought). Error bars denote 95% confidence intervals. Gray shading indicates that the meta‐analysis is based on data sets where less than 85% of county‐level data were included, while black shading indicates that more than 85% of county‐level data were utilized.

Long‐term drought (12‐month) were positively associated with firearm and nonfirearm suicide mortality at all stages of a drought event (Figure 2). The association between drought and suicide presents an inverse U‐shape relationship where the associations are lower at the beginning and end stages of the drought and peak during the most severe conditions of a drought event. A side‐by‐side comparison between firearm and nonfirearm suicides shows that almost all associations are higher for firearm suicides compared to nonfirearm suicides under similar drought conditions. In general, the highest increased risk for firearm suicides was observed during the severe‐to‐exceptional stages, with the largest effect found in the worsening half (IRR: 1.109 [95% CI: 1.091, 1.128]). This was followed by an estimate of 1.094 [95% CI: 1.076,1.112] in the improving half of the drought. The positive association between drought and suicide was slightly lower at the moderate drought conditions toward the end of the drought (IRR: 1.024 [95% CI: 1.014, 1.035]) compared to the moderate drought conditions at the beginning stages of the drought (IRR: 1.031 [95% CI: 1.019, 1.042]). The ARD values are also presented in Figure 2. The highest ARD for firearm suicide was observed in the worsening severe‐to‐exceptional drought conditions with 0.704 [0.595, 0.811] per million persons, followed by improving severe‐to‐exceptional drought with 0.608 [0.501, 0.715] per million persons.

A similar pattern was observed in nonfirearm suicide, although the highest estimate was observed during severe drought during the improving stages (IRR: 1.073 [1.054, 1.093]). The association was lowest at the early stages of the drought (1.017 [0.005,1.029]) and toward the end the positive association remained slightly higher than the early stages (IRR: 1.029 [1.017,1.041]). For nonfirearm suicide, the highest ARD was observed in the improving severe‐to‐exceptional drought conditions with 0.456 [0.344, 0.568] per million persons, followed by worsening severe‐to‐exceptional drought conditions with an ARD of 0.348 [0.233, 0.461] per million persons.

In our statistical analysis, we excluded certain counties because of high standard error. The demographic and drought exposure profiles for these excluded counties showed that they had a median population of 3,246.37 in cases of firearm‐related suicides and 5,480.00 for non‐firearm‐related suicides. For these same counties, the median percentage of time experiencing moderate to severe drought conditions was 10.96% for firearm‐related suicide analysis and 11.84% for non‐firearm‐related suicide analysis. While these insights are specific to the excluded counties, caution is advised as these findings may not be representative of all regions in the United States, particularly the central noncore counties (see Figure S1 in Supporting Information S1).

### 3.3 Association of Drought With Suicide by Age, Sex, and Urbanicity

**Figure 3:**
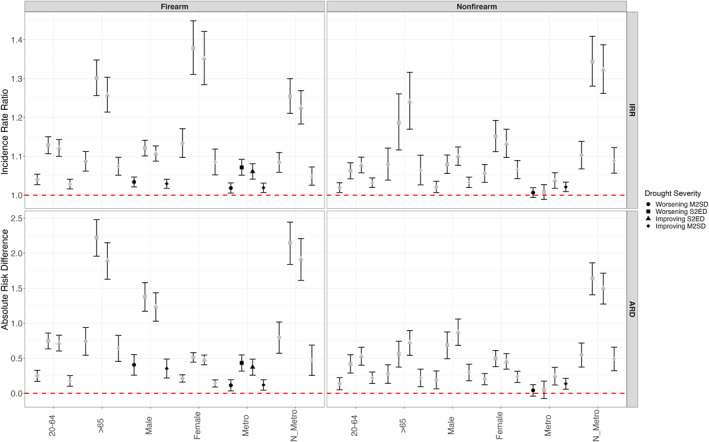
Incidence Rate Ratio (IRR) and the Absolute Risk Difference (per million persons) of firearm and nonfirearm suicides in different drought stages compared to no drought conditions for stratified population based on age, sex and urbanicity (M2SD—moderate‐to‐severe Drought, S2ED—Severe‐to‐Exceptional Drought). Error bars denote 95% confidence intervals. Gray shading indicates that the meta‐analysis is based on data sets where less than 85% of county‐level data were included, while black shading indicates that more than 85% of county‐level data were utilized.

Figure 3 presents IRR and ARD (per million persons) estimates for the stratified assessment by age, sex, and urbanicity. The inverse U‐shape relationship was consistent in the sub‐group analysis with higher associations during the worsening and improving severe‐to‐exceptional drought conditions, and lower associations in worsening and improving moderate‐to‐severe drought conditions (e.g., beginning and end of drought periods) for each population stratum. For brevity, presented estimates in this section are from severe‐to‐exceptional drought phases, although all estimates are shown in Figure 3.

All associations between suicide and drought by age groups were positive, though the associations were strongest during severe phases and among people 65 years or older for both firearm (worsening: 1.301 [1.256,1.347], improving: 1.261 [1.216, 1.306]) and nonfirearm suicides (worsening: 1.187 [1.116,1.261], improving: 1.241 [1.169,1.316]). In general, the positive association was higher in females for both firearm (worsening: 1.383 [1.315, 1.453]) and nonfirearm (worsening: 1.151 [1.111, 1.192]). The association between drought stages and firearm suicides were also significantly positive for both metro and non‐metro counties, though the associations were larger in non‐metro counties and specially in the severe‐to‐exceptional drought conditions for both firearm (worsening: 1.254 [1.209, 1.299], improving: 1.225 [1.183, 1.269]) and nonfirearm mechanisms (worsening: 1.341 [1.278, 1.406], improving: 1.323 [1.262, 1.387]). These results should be interpreted cautiously and may not be generalizable to the entire United States due to the meta‐analysis exclusion criteria causing some geographic regions to be underrepresented. Figure S2 in Supporting Information S1 shows the geographical exclusion is greatest in areas such as the Great Plains and Mountain West.

The stratified ARD estimates in general were an order higher than ARD estimates in the total population, indicating excess burden for specific subgroups. The ARD estimates (in million persons) for age‐based stratification demonstrated a high attribution of drought‐related suicides in older adults (>65) for firearm suicides in both worsening and improving severe‐to‐exceptional drought conditions (worsening: 2.224 [1.959, 2.481], improving: 1.908 [1.643, 2.165]). The ARD estimates also were higher in firearm suicides compared to nonfirearm suicides. In the firearm mechanism, sex‐based stratification showed higher effects in males in all stages of drought compared to females, though the effect was higher in the severe‐to‐exceptional drought conditions (worsening: 1.375 [1.168, 1.578], improving: 1.239 [1.035, 1.441]). In nonfirearm category, the effects on males and females were almost the same. Finally, stratification based on urbanicity for both firearm and nonfirearm types indicates nonmetro counties had a higher number of cases in million persons associated with severe drought conditions than metropolitan counties: Firearm (worsening) 2.142 [1.835, 2.438], (improving) 1.913 [1.609, 2.206]) and nonfirearm (worsening) 1.631 [1.396, 1.853], (improving) 1.501 [1.275, 1.715]).

## 4 Discussion

Our study estimated the association between drought conditions and the incidence of suicide mortality in the United States. We observed that both firearm and nonfirearm suicide incidents were significantly associated with moderate to exceptional drought conditions from 2000 to 2018. Furthermore, there were differences in suicide incidence by drought phase, with a higher incidence of suicide observed during the most severe stages of a drought event and a lower incidence during the more moderate beginning and ending stages of a drought. Another study found a similar pattern where the incidence of mental health issues first increased and then decreased in a non‐linear, inverted U‐shape, corresponding with the severity of the drought (Luong et al., 2021). It was concluded that people experienced heightened psychological distress for the first 2.5–3 years of a drought, after which time this distress dissipates potentially due to acclimation. The rise in suicide during the worsening stages of a drought indicates that individuals are more likely to be affected when the drought persists for a longer period, but as time passes, the level of distress tends to decline as people ultimately adapt to drought and develop mental resilience (Luong et al., 2021; OBrien et al., 2014).

In the total population, the impact of drought was found to be more pronounced on firearm suicides than nonfirearm suicides across all drought conditions; however, these differences should be interpreted with caution, as formal statistical comparisons between subgroup‐specific estimates were not conducted. Suicide mortality risk is associated with suicide method and firearms tend to be a more lethal method (Kennedy et al., 2021; Martínez‐Alés, Gimbrone, et al., 2021; Siegel & Rothman, 2016). Most studies on drought and suicide have been performed in Australian rural and farming communities, where they argue that the heightened risk of suicide is the outcome of multiple factors including, (a) communities more affected physically, psychologically, and economically during drought and (b) firearm availability in the rural and farming population for occupational tasks, such as pest control or protection (Daghagh Yazd et al., 2019; Kennedy et al., 2021; Perceval et al., 2019). We additionally observed that firearm suicide risk is higher in the beginning half as the drought worsens and slightly decreases in the second half, while the nonfirearm suicide risk is higher in the second half of the drought when conditions have started to improve. We hypothesize that in the context of firearm versus nonfirearm suicides, the immediate response to distress may be more pronounced with firearms, given the more lethal and instantaneous nature of this method of suicide (Siegel & Rothman, 2016; Viswanathan et al., 2019; Waldman et al., 2021). It was found by Luong et al. (2021) that the first year of drought could bring more uncertainties and potential risks factors, such as financial insecurity leading to persisting distress (Luong et al., 2021). This underscores the importance of accurate drought forecasting regarding the start, magnitude, and duration of drought events to minimize the unfavorable psychological impacts that occur on different timescales and severities. Better knowledge of drought conditions can optimize times when interventions and mental health education could be most valuable and ensure continuous support during and after the disaster.

We found positive associations between drought and suicide mortality in all adult age groups. Observed differences across age groups should be interpreted as patterns of association rather than statistically tested contrasts. During droughts, working adults often face significant psychological distress due to factors such as financial strain, loss of livelihood, and uncertainty about the future (Hanigan et al., 2012; Viswanathan et al., 2019). They may have limited coping mechanisms and fewer alternative options for employment, making them more vulnerable to the adverse effects of drought on their mental well‐being. Similarly, older populations, particularly those involved in farming, are also more susceptible to the impacts of drought. The aging farming workforce often consists of individuals with limited formal education, higher prevalence of physical health problems, and a reduced ability to adapt to rapidly changing regulatory and technological trends in the global farming market (Kennedy et al., 2021). These factors, along with the greater availability of firearms, contribute to the increased vulnerability of older individuals during droughts.

Our study also showed that both firearm and nonfirearm suicide risks were higher in females compared to males, especially in severe drought conditions, though there was lower certainty due to the smaller suicide sample size of this subgroup. Women often face unique social and economic challenges during drought periods, which can exacerbate stress and anxiety levels. As primary caregivers, women may experience increased responsibilities in managing limited water resources, ensuring the well‐being of their families, and coping with the potential loss of livelihoods (Hanigan et al., 2018; Perceval et al., 2019). Additionally, the psychological impact of drought, such as financial strain, food insecurity, and displacement, can disproportionately affect women, leading to higher levels of distress. Furthermore, existing gender inequalities and societal norms may restrict women's access to mental health resources, preventing timely intervention and support. A few studies on drought and mental health support these findings; Hanigan et al. (2018) showed drought was associated with higher distress in younger rural women (aged 40–54). Another case study in Northern Brazil concludes that in the drought area, women had significantly higher levels of anxiety and men had significantly higher levels of emotional distress (Coêlho et al., 2004).

While our study found a higher association between drought conditions and suicide among females, the measures of absolute risk difference (ARD) are more elevated among males. This discrepancy can be attributed to the fact that the baseline suicide mortality rate used to calculate the ARD is intrinsically higher in males compared to females. This suggests that while both sexes are affected by drought‐related mental health challenges, males may be more likely to resort to suicide as a response to the distress caused by severe drought conditions.

Finally, in this study, we showed that the association between drought and suicide is stronger in non‐metro counties both for firearm and nonfirearm suicides. As with other subgroup findings, these rural–urban differences are descriptive and were not statistically compared. This finding is aligned with majority of the studies on drought and mental distress (Berman et al., 2021; Kennedy et al., 2020; Santos et al., 2021; Waldman et al., 2021). Drought and mental health are closely linked in rural areas. Prolonged dry spells associated with drought can result in crop failures and financial strain for farmers, leading to stress and anxiety (Berman et al., 2021). The harsh living conditions brought on by drought can also lead to feelings of hopelessness and depression. Vins et al. (2015) in a systematic review on drought and mental health concluded that financial strains on farming families can seriously deteriorate mental health, even when partially offset by welfare payments for drought relief. In addition, it was shown that droughts accelerate emigration from afflicted areas, weakening social networks and reducing social connections, which may have an influence on mental health due to the ongoing loss of regional hospitals, schools, and industries. Perceived stigma about help‐seeking, precarious access to physical and mental health providers, lack of education, unemployment, higher exposure to pesticides and agrochemicals specially in farming communities, geographic isolation, poverty, and complex financial arrangements are among the risk factors for suicides in rural areas (Berman et al., 2021). Waldman et al. (2021) showed that the well‐being of farmers has a ripple effect on rural communities, regardless of occupation, implying that both farmers and non‐farmers alike experience the effects of declining trends in the farm economy.

Due to reduced rainfall, high temperature, and high evaporation, severe drought will often occur in conjunction with other environmental and societal stressors, creating a complex and challenging situation for affected communities. Longer and more severe drought can be compounded by heatwaves, wildfires, and economic hardships, exacerbating the negative impacts on health, agriculture, and other sectors. Summer heatwaves, especially in farming communities, have been associated with farmers distress due to multiple factors including, but not limited to long hours at work, crop loss, and change of landscape (Daghagh Yazd et al., 2019; Deisenhammer et al., 2003; Hanigan et al., 2012; Singh et al., 2015). Since drought is an underpinning of many extreme environmental conditions, it is important to attribute drought as an umbrella cause of adverse mental health conditions from the environment.

There is much work to be done to better understand the mechanisms that lead to increased suicide during drought conditions. An improved causal framework will support the development of interventions to reduce drought impacts and assist behavioral health experts in addressing these challenges. As drought conditions are projected to become more severe and intense in many parts of the world under climate change, it is important to identify these relationships to build better public health and adaptive strategies.

Our study's conclusions are subject to several limitations. Primarily, although numerous drought indices exist, our analysis was confined to one—the EDDI which we converted into USDM categories. Future research should explore alternative drought indices and compare them to EDDI to assess whether findings are robust across metrics. Additionally, our ecological study design did not allow for individual‐level risk assessment, nor could it adjust for personal confounders such as education level. Given that suicide is influenced by an array of psychological, social, biological, occupational, and environmental factors, our inability to consider these elements presents a significant limitation. Future studies could use individual‐level data or longitudinal cohort designs to better capture these dimensions. Moreover, critical risk factors for at‐risk populations, including occupational hazards, pesticide exposure, agricultural practices, and state‐specific gun policies, were beyond our study's scope. A further constraint was the statistical power; the rarity of suicide events coupled with the small populations in many counties limited our capacity to analyze certain subgroups, particularly those aged 0–19 and racial demographics. Caution is necessary when interpreting several of our estimates, as they excluded multiple counties from the meta‐analysis due to high statistical uncertainties. IRRs and ARDs stratified by demographic groups and urbanicity reflect the geographic regions illustrated in the maps in the supplementary materials. Often, counties in the Midwest region of the United States, especially in Nebraska, Kansas, North Dakota, and South Dakota, were excluded. This was due to their small populations, insufficient suicide death counts, and less variability in drought categories. They also more heavily represent counties in California and the Southwest regions of the United States that have large populations and a greater prevalence of drought.

## 5 Conclusion

Our ecological time‐series study suggests that almost all drought stages from moderate to exceptional drought were associated with a heightened risk of firearm and nonfirearm suicides from 2000 to 2018 in the contiguous United States. Associations were observed across population subgroups, with higher estimated effects among older adults, females, and rural communities, warranting cautious interpretation in the absence of formal interaction testing. This study provides first‐time evidence of increased drought and suicide risk to certain U.S. sub‐populations and supports the need for incorporating mental health into drought early warning plans and local mitigation strategies to reduce the burden on population.

## Conflict of Interest

The authors declare no conflicts of interest relevant to this study.

## Supporting information

Supporting Information S1

## Data Availability

The Evaporative Demand Drought Index (EDDI) used to characterize drought exposure is publicly available through NOAA's Physical Sciences Laboratory (NOAA Physical Sciences Laboratory, n.d.). Monthly temperature data were obtained from the NOAA Monthly U.S. Climate Gridded Data set (NClimGrid) (Vose et al., 2014). Mortality data were obtained from the National Center for Health Statistics (NCHS) Multiple Cause of Death (MCOD) restricted‐use files, which are not publicly available due to confidentiality protections and require an approved data use agreement; information on accessing these data is provided by NCHS (National Center for Health Statistics, n.d.‐a; National Center for Health Statistics, n.d.‐b). County‐level urban–rural classifications were obtained from the NCHS 2013 Urban–Rural Classification Scheme for Counties (National Center for Health Statistics, n.d.‐a; National Center for Health Statistics, n.d.‐b). All analysis code used to process data, conduct statistical analyses, and generate figures is publicly available and preserved in a trusted repository (Abadi, 2026). Statistical analyses were conducted using *R* (version 4.4.1), including the mgcv and metafor packages.
