# Supplementary material for: The Lethal Connection: Investigating the Relationship of Drought Conditions on Firearm and Nonfirearm Suicides Among U.S. Adults
Source: Geohealth. 2026 May 19;10(5):e2025GH001571. doi: 10.1029/2025GH001571 (PMC13184638; doi:10.1029/2025GH001571)
Supplement: Supplementary file 1 — Supporting Information S1 [file GH2-10-e2025GH001571-s001.docx]

Supplementary Material

**The Lethal Connection: Investigating the Relationship of Drought Conditions on Firearm and Nonfirearm Suicides among U.S. Adults**

**Azar M. Abadi^1^, Yeongjin Gwon ^2^, Melissa J. Smith ^3^, Jesse D. Berman^4^, Austin Rau^4^, Ronald D. Leeper^5^, Jared Rennie^6^, Siddhi Munde^7^, Babak J. Fard^7^, Jesse E. Bell^7,8,9^**

^1^Department of Environmental Health Sciences, School of Public Health, University of Alabama at Birmingham, Birmingham, AL, USA

^2^Department of Biostatistics, College of Public Health, University of Nebraska Medical Center, Omaha, NE, USA

^3^Department of Biostatistics, School of Public Health, University of Alabama at Birmingham, Birmingham, AL, USA

^4^Division of Environmental Health Sciences, School of Public Health, University of Minnesota, Minneapolis, MN, USA

^5^North Carolina Institute for Climate Studies, North Carolina State University, Raleigh, NC, USA

^6^NOAA’s National Centers for Environmental Information, Asheville, NC, USA

^7^Department of Environmental Agriculture Occupational and Health, College of Public Health, University of Nebraska Medical Center, Omaha, NE, USA

^8^University of Nebraska, Daugherty Water for Food Global Institute, Lincoln, NE, USA

^9^University of Nebraska-Lincoln, School of Natural Resources, Lincoln, NE, USA

Corresponding author: Azar M. Abadi ([aabadi@uab.edu](mailto:aabadi@uab.edu))

Supporting Information – Table of Contents

Table S1. Comparative incidence rate ratios of suicide associated with 6-month and 12-month drought timescales, stratified by suicide method (firearm vs. non-firearm), drought category, and geographic coverage.

Table S2. Sensitivity analyses of inclusion criteria for the meta-analysis examining the association between drought and firearm suicide (12-month exposure), including alternative population-based thresholds.

Figure S1. Geographic distribution of counties excluded from firearm (a) and non-firearm (b) suicide analyses based on the standard error > 2 exclusion criterion.

Figure S2. Geographic distribution of counties excluded from firearm (a) and non-firearm (b) suicide analyses across demographic strata (age > 65, female, and non-metro).

Table S1. Comparative Incidence Rate Ratios of suicide associated with 6-month and 12-month drought timescales, categorized by suicide method (Firearm vs. Non-Firearm). For each IRR, the reference category is no drought. This table also includes the geographical coverage to demonstrate the generalizability of the findings across the US.

| **Duration** | **6-month** | **Suicide Type** | **Drought Category** | **IRR and 95% CI** | **P value** | **Geographical Coverage** |
| --- | --- | --- | --- | --- | --- | --- |
|  |  | Firearm | Moderate to Severe Worsening | 1.030 (1.018,1.042) | <0.0001 | Represents over 85% of counties |
|  |  |  | Severe to Exceptional Worsening | 1.105 (1.085,1.126) | <0.0001 | Represents under 85% of counties |
|  |  |  | Severe to Exceptional Improving | 1.077 (1.061,1.094) | <0.0001 | Represents under 85% of counties |
|  |  |  | Moderate to Severe Improving | 1.033 (1.022,1.045) | <0.0001 | Represents over 85% of counties |
|  |  | Non-Firearm | Moderate to Severe Worsening | 1.028 (1.015,1.040) | <0.0001 | Represents under 85% of counties |
|  |  |  | Severe to Exceptional Worsening | 1.062 (1.042,1.083) | <0.0001 | Represents under 85% of counties |
|  |  |  | Severe to Exceptional Improving | 1.049 (1.032,1.067) | <0.0001 | Represents under 85% of counties |
|  |  |  | Moderate to Severe Improving | 1.018 (1.005,1.031) | 0.005 | Represents under 85% of counties |
|  | **12-month** | Firearm | Moderate to Severe Worsening | 1.032 (1.020,1.043) | <0.0001 | Represents over 85% of counties |
|  |  |  | Severe to Exceptional Worsening | 1.109 (1.091,1.128) | <0.0001 | Represents under 85% of counties |
|  |  |  | Severe to Exceptional Improving | 1.093 (1.076,1.112) | <0.0001 | Represents under 85% of counties |
|  |  |  | Moderate to Severe Improving | 1.025 (1.014,1.035) | <0.0001 | Represents over 85% of counties |
|  |  | Non-Firearm | Moderate to Severe Worsening | 1.018 (1.007,1.030) | 0.002 | Represents under 85% of counties |
|  |  |  | Severe to Exceptional Worsening | 1.057 (1.037,1.077) | <0.0001 | Represents under 85% of counties |
|  |  |  | Severe to Exceptional Improving | 1.074 (1.055,1.093) | <0.0001 | Represents under 85% of counties |
|  |  |  | Moderate to Severe Improving | 1.030 (1.019,1.042) | <0.0001 | Represents under 85% of counties |

**Table S2.** Sensitivity analysis of inclusion criteria for the meta-analysis examining the association between drought and firearm suicide (12-month exposure). Results are shown for two drought constructs—M2SDImp (intensifying droughts) and M2SDWrs (worsening droughts)—under varying county-level inclusion criteria. The primary criterion used in the final analysis (standard error ≤ 2) excluded approximately 11–13% of counties. Findings were consistent across alternative population-based thresholds (≥10k, ≥25k, ≥30k), supporting the robustness of the results.

| **Inclusion Criterion** | **Drought Type** | **n total** | **n Excluded** | **% Excluded** | **IRR** | **95% CI** |
| --- | --- | --- | --- | --- | --- | --- |
| SE ≤ 2 | M2SDImp | 3107 | 341 | 10.975% | 1.024 | (1.013, 1.035) |
| Pop ≥ 10,000 | M2SDImp | 3107 | 952 | 30.641% | 1.019 | (1.009, 1.031) |
| Pop ≥ 25,000 | M2SDImp | 3107 | 1860 | 59.864% | 1.018 | (1.006 ,1.029) |
| Pop ≥ 30,000 | M2SDImp | 3107 | 2057 | 66.205% | 1.018 | (1.006, 1.030) |
| SE ≤ 2 | M2SDWrs | 3107 | 392 | 12.616% | 1.031 | (1.019, 1.043) |
| Pop ≥ 10,000 | M2SDWrs | 3107 | 952 | 30.641% | 1.026 | (1.014, 1.037) |
| Pop ≥ 25,000 | M2SDWrs | 3107 | 1860 | 59.864% | 1.022 | (1.009, 1.034) |
| Pop ≥ 30,000 | M2SDWrs | 3107 | 2057 | 66.205% | 1.021 | (1.008, 1.033) |

| a) |
| --- |
| 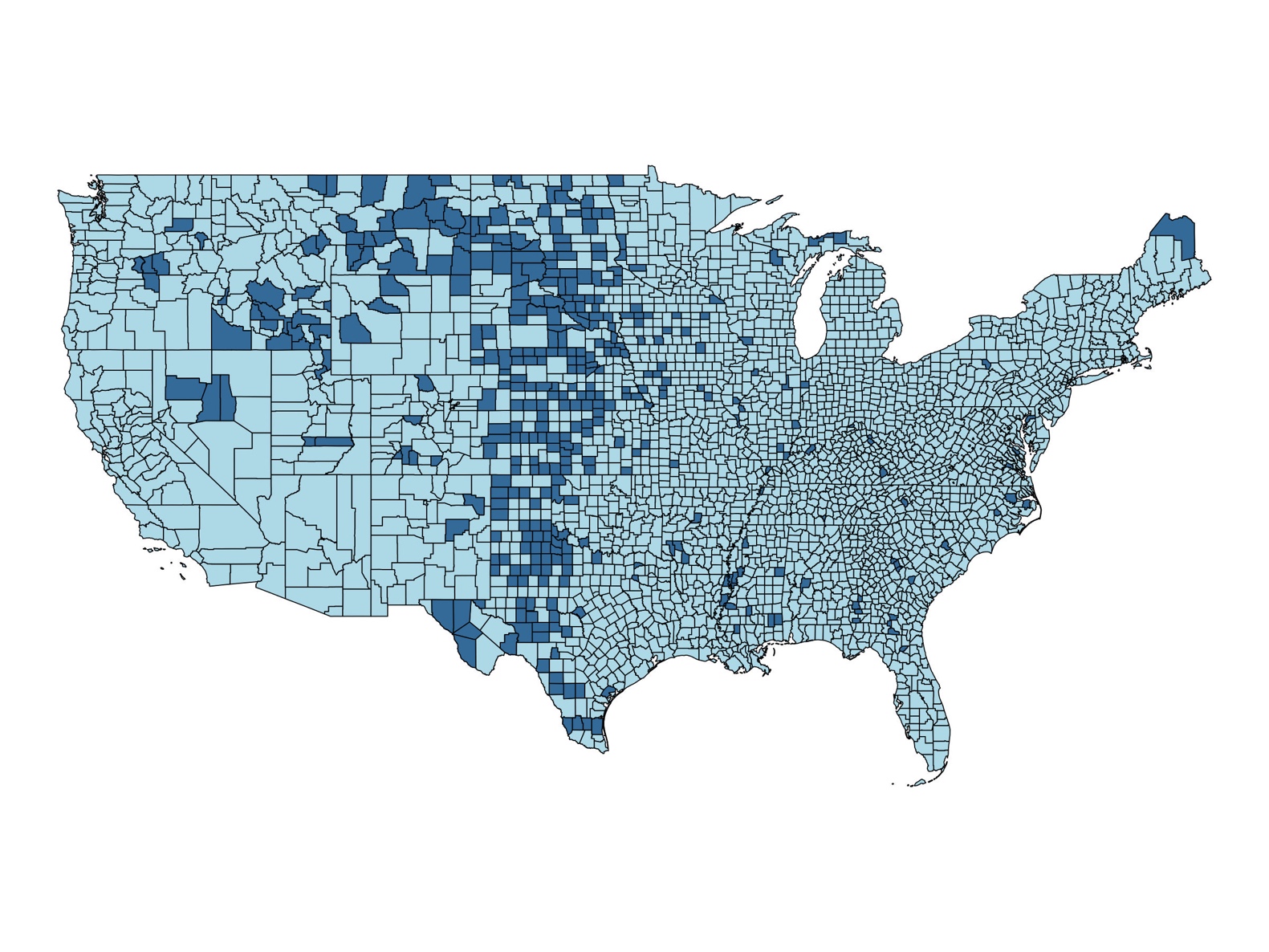 |
| b) |
| 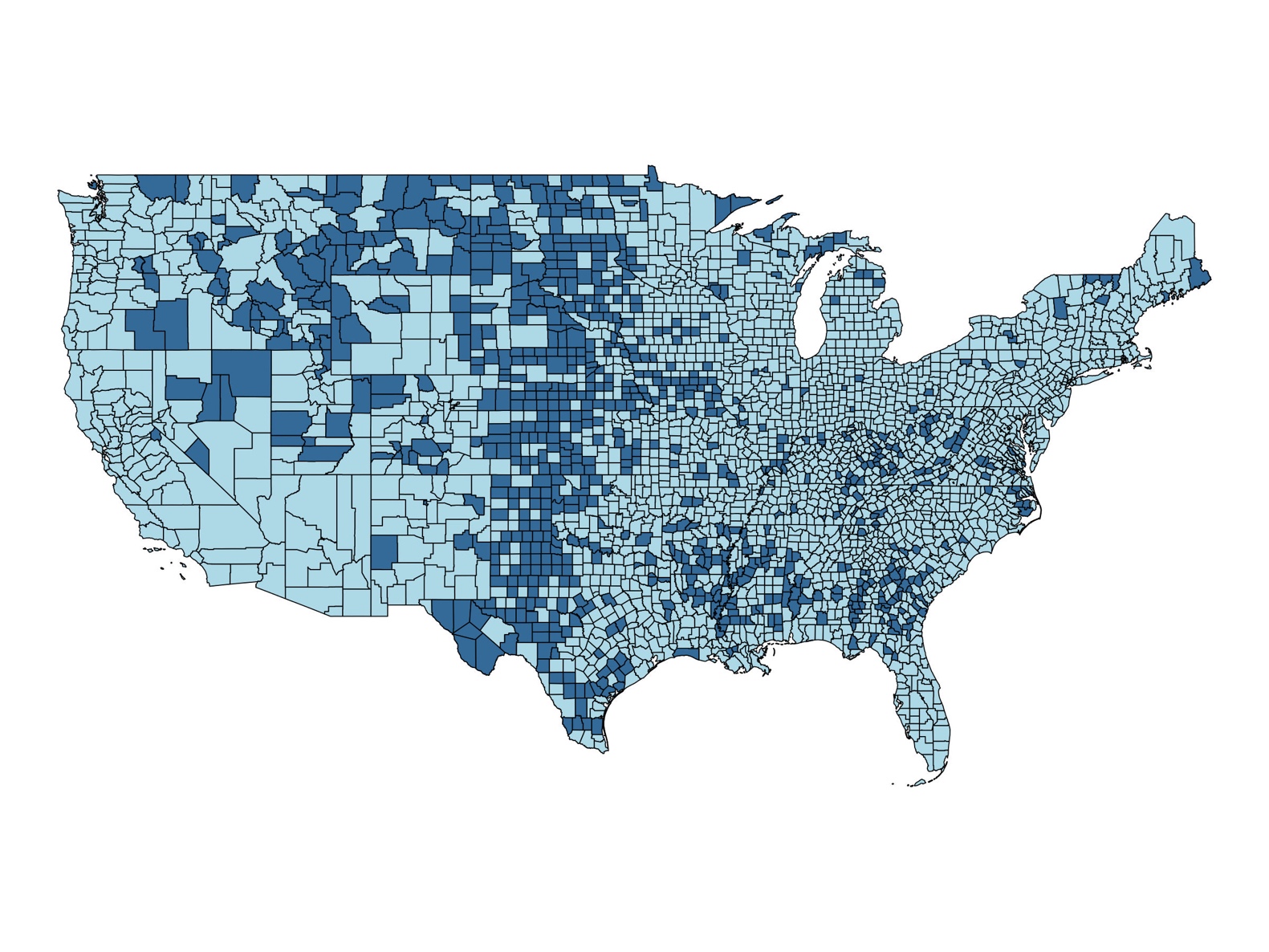 |

Figure S1. Geographical Representation of Excluded Counties in Firearm (a) and Non-Firearm Suicide (b) Analyses: Shown here are the counties in dark blue that were excluded from the meta-analysis examining the association between moderate to severe drought and suicides across the U.S., based on the exclusion criterion of a standard error exceeding 2. The spread of these counties serves as an indicator of where IRR estimates were stable and unstable, providing context for the robustness and limitations of our model in these specific regions.

| a) Firearm | b) Non-firearm |
| --- | --- |
| Age > 65 | |
| 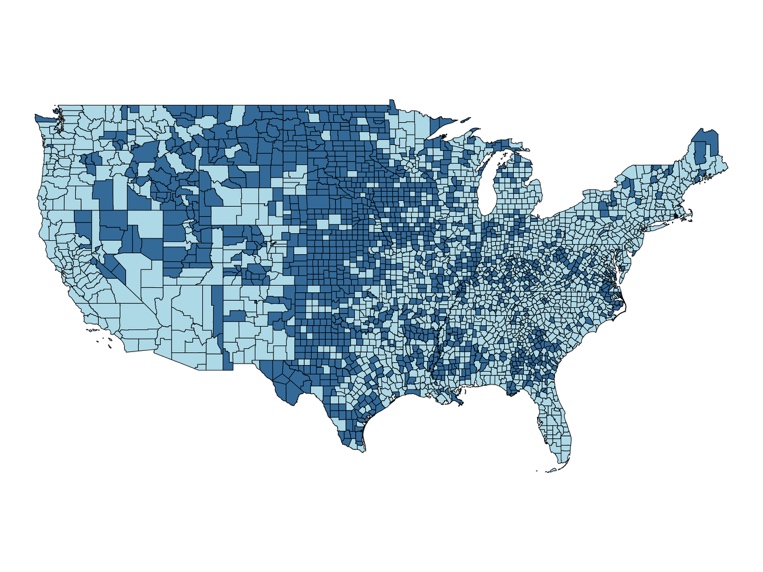 | 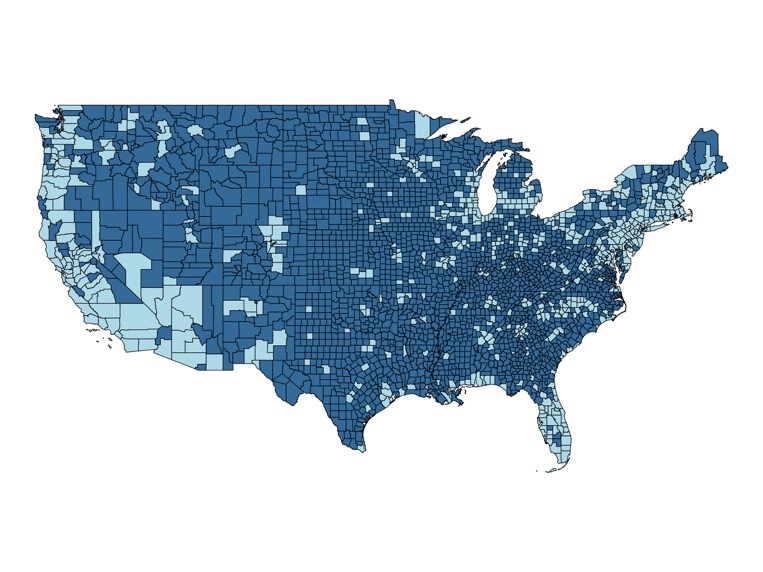 |
| Female | |
| 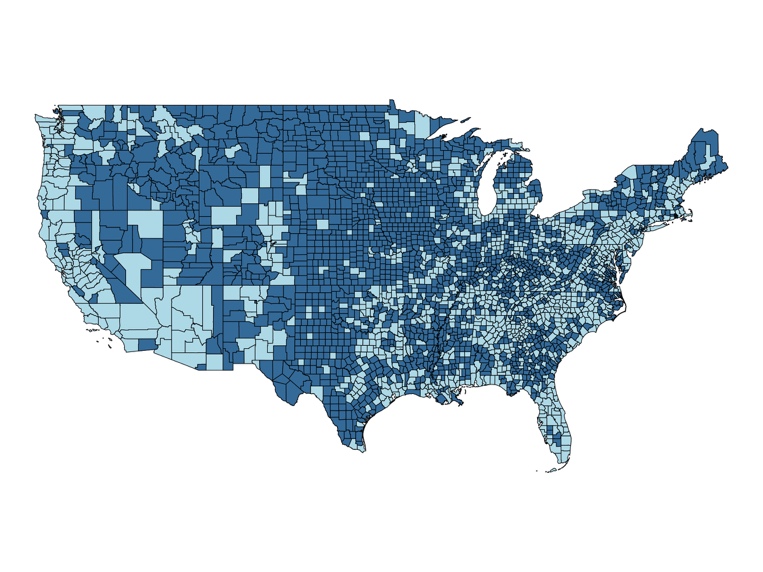 | 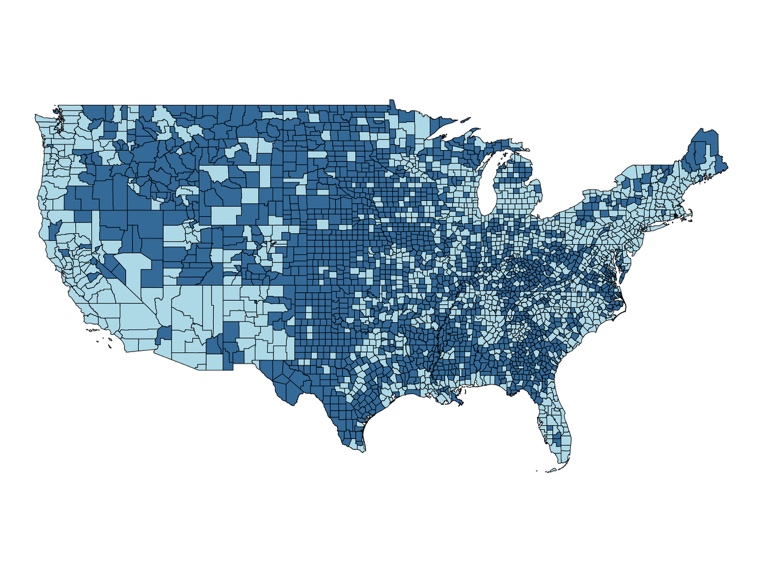 |
| Non-metro | |
| 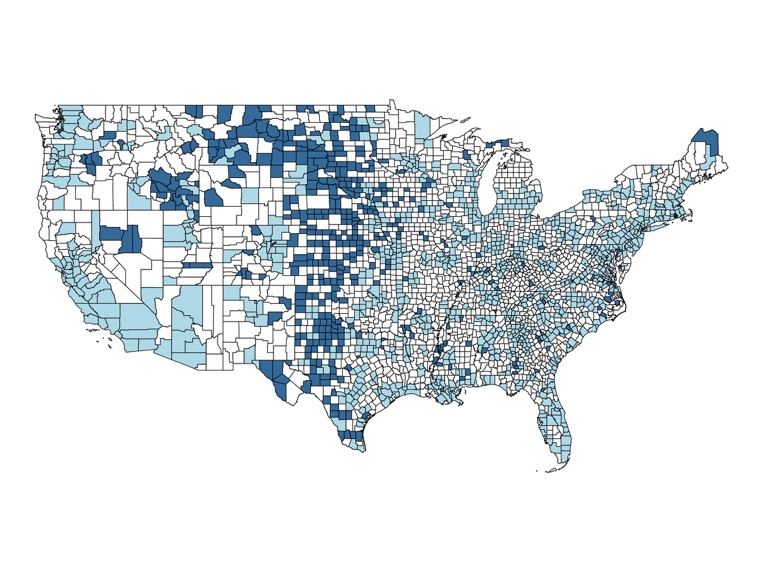 | 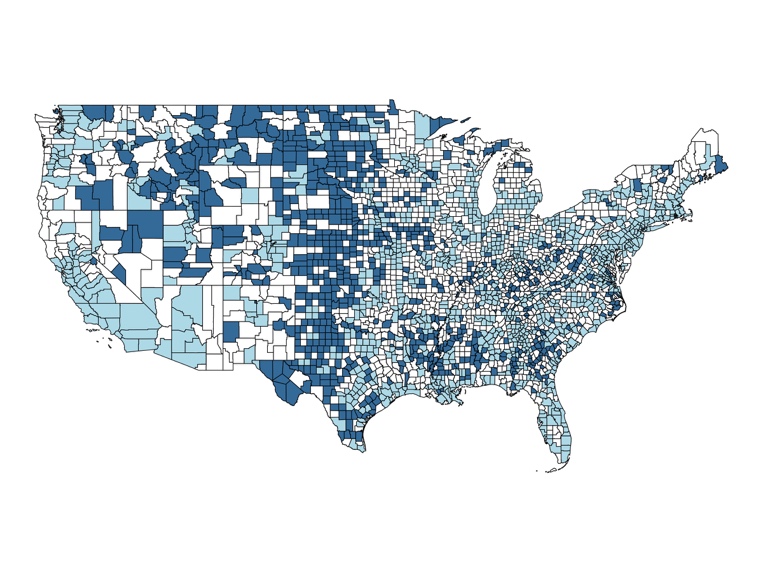 |

Figure S2. Geographical Representation of Excluded Counties in Firearm (a) and Non-Firearm Suicide (b) Analyses: Shown here are the counties that were excluded from the meta-analysis examining the association between moderate to severe drought and suicides across the U.S., based on the exclusion criterion of a standard error exceeding 2. In all panels, dark blue indicates the counties that were excluded from the analysis. The last two maps in the non-metro category differentiate between all non-metro counties (shown in white) and the excluded non-metro counties (marked in dark blue). The spread of these counties serves as an indicator of where IRR estimates were stable and unstable, providing context for the robustness and limitations of our model in these specific regions.
